# *Ancylostoma ceylanicum* Hookworm, Rural Papua New Guinea, 2020

**DOI:** 10.3201/eid3207.251657

**Published:** 2026-07

**Authors:** Jessica L. Scott, Daniel Pelowa, Wayne Melrose, Jeffrey M. Warner, Catherine M. Rush

**Affiliations:** James Cook University College of Medicine and Dentistry, Townsville, Queensland, Australia (J.L. Scott, W. Melrose, J.M. Warner, C.M. Rush); James Cook University Australian Institute of Tropical Health and Medicine, Townsville (J.L. Scott, J.M. Warner, C.M. Rush); Balimo District Hospital, Balimo, Papua New Guinea (D. Pelowa)

**Keywords:** hookworm, parasites, Ancylostoma ceylanicum, Necator americanus, Ancylostoma, zoonoses, Papua New Guinea

## Abstract

We conducted a cross-sectional study of zoonotic hookworm *Ancylostoma ceylanicum* in humans in Western Province, Papua New Guinea, confirmed by internal transcribed spacer sequencing. Overall hookworm prevalence was 54.9%; *A. ceylanicum* hookworms were present in 3.3% of specimens. One Health approaches are needed for hookworm control in Papua New Guinea.

Hookworm infections pose a major public health challenge in Papua New Guinea (PNG). Historic national estimates suggest that up to three quarters of the population may be infected (*1*); a 2025 study reported prevalence >80% for the anthropophilic hookworm species *Necator americanus* in Madang Province (*2*). In 2018, the zoonotic hookworm *Ancylostoma ceylanicum* was confirmed in a migrant worker returning from Manus Island by using molecular methods (*3*). However, since that initial report, no data have been published on the prevalence of *A. ceylanicum* hookworm among local populations in PNG. We report molecular evidence of locally acquired *A. ceylanicum* infections in a rural community in PNG, alongside a high overall prevalence of hookworm infection. 

We conducted a cross-sectional study in collaboration with the Balimo District Hospital (Balimo, PNG) during January 2020. We recruited community members >16 years of age through convenience sampling from Balimo, which is situated in the Delta Fly District of the Western Province of PNG (Appendix Figure 1). We preserved fecal specimens in sodium-acetate 5% formalin (SAF) and separately in guanidinium thiocyanate within 8 hours of submission. We examined SAF-preserved specimens for hookworm ova by microscopy, using direct unconcentrated and ethyl acetate–concentrated fecal smears.

We extracted genomic DNA from guanidinium thiocyanate–preserved fecal specimens using the Zymo Quick-DNA Fecal/Soil Microbe Miniprep Kit (Zymo Research Corporation, https://www.zymoresearch.com). We detected 3 hookworm species, *N. americanus*, *A. ceylanicum,* and *A. duodenale*, using TaqMan quantitative PCR (qPCR) (Integrated DNA Technologies, https://sg.idtdna.com) (Appendix Table). We Sanger sequenced all *Ancylostoma* qPCR-positive samples targeting the internal transcribed spacer region, using custom primers (forward 5′-GAATGCCGCCTTACTGCTTG-3′ and reverse 5′-CGATTCAGCAGCAACAACGAG-3′) (Appendix).

Among the 122 participants who submitted a fecal specimen, microscopy detected hookworm ova in 28 (22.9%), whereas qPCR identified 64 (52.5%) as positive. Combining both methods yielded an overall hookworm prevalence of 54.9% (67/122) (Table). Three (4.5%) samples that tested positive by microscopy were negative by qPCR. *N. americanus* was the predominant hookworm species detected by qPCR, identified in 64 (52.5%) participants. We detected *Ancylostoma* spp. hookworm in 4 (3.3%) samples; all *Ancylostoma*-positive participants were also infected with *N. americanus*. All sequences identified in this study (GenBank accession nos. PV530493–6) formed a distinct cluster with *A. ceylanicum* sequences, including the positive template control isolate (accession no. PV530497) and the PNG isolate previously identified from the migrant worker (accession no. LC036567), confirming all 4 cases as *A. ceylanicum* hookworm (Figure). We detected no *A. duodenale* cases (Appendix Figure 2).

**Table:** Prevalence of hookworm infections found in study of *Ancylostoma ceylanicum* hookworm, rural Papua New Guinea, 2020*

Method	No. (%)		
	Hookworm	*Necator americanus*	*Ancylostoma* spp.
Microscopy	28 (22.9)	NA	NA
TaqMan qPCR†	64 (52.5)	64 (52.5)	4 (3.3)
Total	67 (54.9)	64 (52.5)	4 (3.3)

*NA, not applicable; qPCR, quantitative PCR. †Integrated DNA Technologies, https://sg.idtdna.com.

**Figure:**
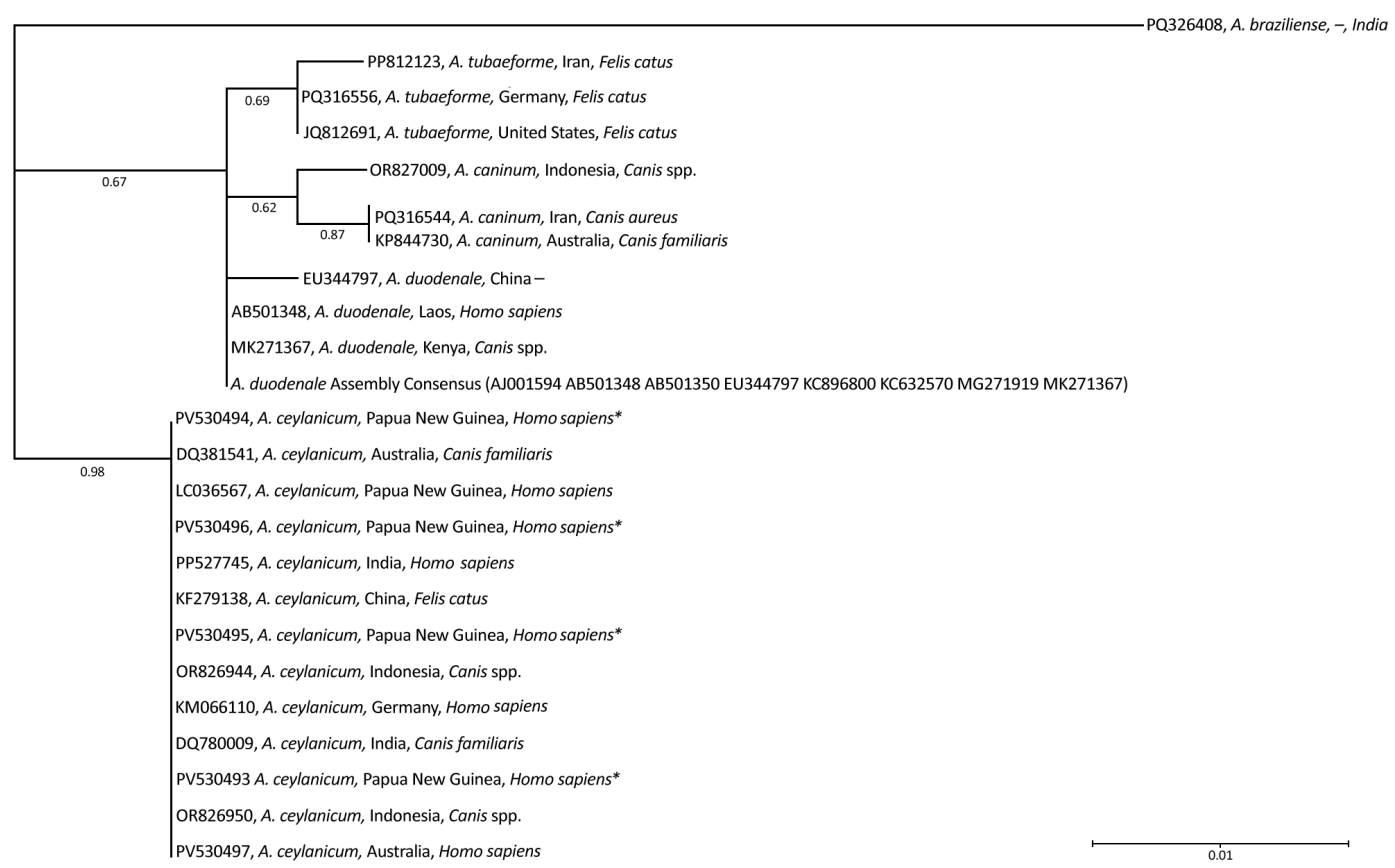
Phylogenetic analysis of *Ancylostoma* spp. in study of *A. ceylanicum* hookworm, rural Papua New Guinea, 2020. Analysis is based on the internal transcribed spacer sequence regions 1 and 2 and conducted with the maximum-likelihood method, using the Kimura 2-parameter model. Model selection was based on penalized-likelihood information criteria. The phylogenetic tree was visually adjusted using Interactive Tree of Life version 7.2.2 (https://itol.embl.de). Values at branch nodes indicate bootstrap support values (1,000 replicates). *A. braziliense* was used as an outgroup to root the tree. GenBank accession numbers are shown; asterisks (*) denote sequences identified in this study (accession nos. PV530493–96). We used *A. ceylanicum* (accession no. PV530497) as a positive control. Dash (–) indicates that no information was available for a particular attribute of the strain. Scale bar represents 0.01 substitutions per site.

This study provides molecular evidence of *A. ceylanicum* hookworm in a rural community in Western Province, PNG. Our findings further support the recognition of *A. ceylanicum* as the second most common human hookworm in the Asia-Pacific region (*4*); however, in our study it accounted for only a small proportion of the overall hookworm burden compared with *N. americanus*.

The absence of *A. duodenale* hookworm prompts reconsideration of earlier hookworm surveys in PNG. Previous studies relied on larval culture for species identification; however, the close morphologic similarity between *A. duodenale* and *A. ceylanicum* larvae might have led to misclassification, raising the possibility that *A. ceylanicum* infections were historically present but attributed to *A. duodenale*. Whether that is the case or that *A. ceylanicum* hookworm was more recently introduced remains unclear. A study in Madang Province found neither *A. ceylancium* nor *A. duodenale* hookworm despite high hookworm prevalence, indicating geographic variation could also exist (*2*). Conditions in Balimo, including free-roaming dogs that serve as household guardians, may favor potential zoonotic transmission of *A. ceylanicum* hookworms. At the time of our study, veterinary services and deworming programs were absent, creating opportunities for parasite persistence at the human–animal–environment interface. Nevertheless, without concurrent sampling of local dogs and cats, the contribution of animal reservoirs and the directionality of transmission remain unclear.

Delayed specimen processing might have resulted in lysis of hookworm ova, thereby reducing the sensitivity of microscopy in our study. Furthermore, hookworm ova are morphologically similar to those of *Strongyloides fuelleborni* subspecies *fuelleborni*, which is known to occur in the region (*5*); strongyloidiasis has been detected in the same community in which we conducted our study (*6*). That overlap in egg morphology might represent one of several factors contributing to the discrepancy observed between microscopy-positive and qPCR-negative specimens. In addition, the modest sample size and convenience sampling of participants >16 years of age limit the generalizability of our findings to the wider community and surrounding areas. Given that untreated hookworm infection can persist long term, prevalence estimates may also be influenced by chronic infections within the sampled population.

In summary, the presence of *A. ceylanicum* hookworms in this region requires a One Health approach to addressing both human infections and animal reservoirs. Traditional deworming programs targeting only humans are insufficient for preventing reinfection and achieving sustainable elimination where zoonotic transmission may occur.

Appendix Additional information from study of *Ancylostoma ceylanicum* hookworm, rural Papua New Guinea, 2020.
