# Supplementary material for: Ancylostoma ceylanicum Hookworm, Rural Papua New Guinea, 2020
Source: Emerg Infect Dis. 2026 Jul;32(7):1217–9. doi: 10.3201/eid3207.251657 (PMC13322408; doi:10.3201/eid3207.251657)
Supplement: Appendix — Additional information from study of Ancylostoma ceylanicum hookworm, rural Papua New Guinea, 2020. [file 25-1657-Techapp-s1.pdf]

Article DOI: https://doi.org/10.3201/eid3207.251567

EID cannot ensure accessibility for supplementary materials supplied by authors. Readers who have difficulty accessing supplementary content should contact the authors for assistance.

Ancylostoma ceylanicum Hookworm, Rural Papua New Guinea, 2020

Appendix

DNA Extraction and Reaction Conditions of the TaqMan qPCRs for the Detection of Hookworm

Genomic DNA was extracted from fecal specimens stored in GTC, using the Zymo-Quick-DNA fecal/soil kit microbe miniprep kit (Zymo-research Corporation, Irvine, CA). DNA was isolated from the fecal specimen as per manufacturer instructions, but the bead beating step was modified to one cycle, for 45 seconds on the FastPrep-24 (MP Biomedicals, USA) at full speed (6.5 m/s). All qPCR reactions in this study were performed as a singleplex. Positive template DNA controls used were based on specimens confirmed to have *N. americanus* and *A. ceylanicum* (kindly provided by Professor Alex Loukas, James Cook University (JCU), Queensland, Australia). All extracted fecal DNA specimens were used in optimised TaqMan qPCR reactions performed using 10µL of the GoTaq qPCR Probe Master Mix (Promega) with 2µL of stool-derived DNA in a final reaction volume of 20µL. Cycling conditions for the TaqMan qPCR consisted of the following parameters: one cycle of 95°C for five minutes; followed by 40 cycles of 95°C for 10 seconds and 60°C for 60 seconds, performed on the Bio-Rad CFX96 Touch thermocycler. A non-template control (nuclease-free water) and stool specimen known to be negative for all STHs were included in each run as negative controls.

Ancylostoma spp. Primer Design for Sequencing and Cycle Conditions

Custom primers (Forward 5′-GAATGCCGCCTTACTGCTTG-3′ and Reverse 5′-CGATTCAGCAGCAACAACGAG-3′) designed in Geneious Prime (v.2024.0.5) were based on a previously published primer set (*1*), but were designed to target a larger, 434-bp (bp) region of *Ancylostoma* species. These primers were designed against sequences belonging to *A. duodenale*

(GenBank accession numbers: EU344797, MK271367), *A. caninum* (GenBank accession numbers: KP0844730, PQ316544, OR827009), and *A. ceylanicum* (GenBank accession numbers: LC036567, DQ381541, KF279138, KM066110, OR826944).

Reactions were performed with 10µL Master mix (GoTaq® Probe qPCR Kit, Promega), 2µL of DNA template, then 0.5µM of each primer, prepared to a final reaction volume of 20µL. Reactions were performed using the SimpliAmp thermocycler under the following conditions: one cycle of 95°C for five minutes, followed by 40 cycles of 95°C for 30 seconds, 64°C for 30 seconds and 72°C for 30 seconds, then a final extension held at 64°C for 7 minutes. PCR products were verified using gel electrophoresis and were then cleaned (Wizard SV Gel and PCR Clean-Up System; Promega) before being sequenced at the Australian Genomics Research Facility, Brisbane, Queensland, Australia.

References

1. Traub RJ, Inpankaew T, Sutthikornchai C, Sukthana Y, Thompson RCA. PCR-based coprodiagnostic tools reveal dogs as reservoirs of zoonotic ancylostomiasis caused by *Ancylostoma ceylanicum* in temple communities in Bangkok. Vet Parasitol. 2008;155:67–73. PubMed https://doi.org/10.1016/j.vetpar.2008.05.001

2. Verweij JJ, Brienen EA, Ziem J, Yelifari L, Polderman AM, Van Lieshout L. Simultaneous detection and quantification of *Ancylostoma duodenale*, *Necator americanus*, and *Oesophagostomum bifurcum* in fecal samples using multiplex real-time PCR. Am J Trop Med Hyg. 2007;77:685–90. PubMed https://doi.org/10.4269/ajtmh.2007.77.685

3. Hii SF, Senevirathna D, Llewellyn S, Inpankaew T, Odermatt P, Khieu V, et al. Development and evaluation of a multiplex quantitative real-time polymerase chain reaction for hookworm species in human stool. Am J Trop Med Hyg. 2018;99:1186–93. PubMed https://doi.org/10.4269/ajtmh.18-0276

Appendix Table. Singleplex TaqMan qPCR target sequences for hookworm, primers and probe concentrations used in study*

Target	Primer/ probe	Sequence (5′→3′)	Size, bp	Gene target	Final conc, nM	Reference
Necator americanus	Forward	CTGTTTGTCGAACGGTACTTGC	101	ITS2	100	Verweij, et al. (*2*)
	Reverse	ATAACAGCGTGCACATGTTGC			100	
	Probe	CY5-CTG[+T]A[+C]TA[+C]G[+C]AT[+T]GTATAC-IBRQ*			50	
Ancylostoma spp.	Forward	CGGGAAGGTTGGGAGTATC	104	ITS1	100	Hii, et al. (*3*)
	Reverse	CGAACTTCGCACAGCAATC			100	
A. ceylanicum	Probe	HEX-CCGTTC[+C]TGGGTGGC-IBFQ†			50	
A. duodenale	Probe	FAM-TCGTTAC[+T][+G]GGTGACGG-IBFQ†			50	

*Bp, base pairs; BHQ, black hole quencher; IBRQ, Iowa black RQ quencher; IBFQ, Iowan black FQ quencher; ITS, internal transcribed spacer.
†Modifications made to the probe reporters and/or quenchers. Probe oligonucleotides with [+] denote locked nucleic acids (LNAs).

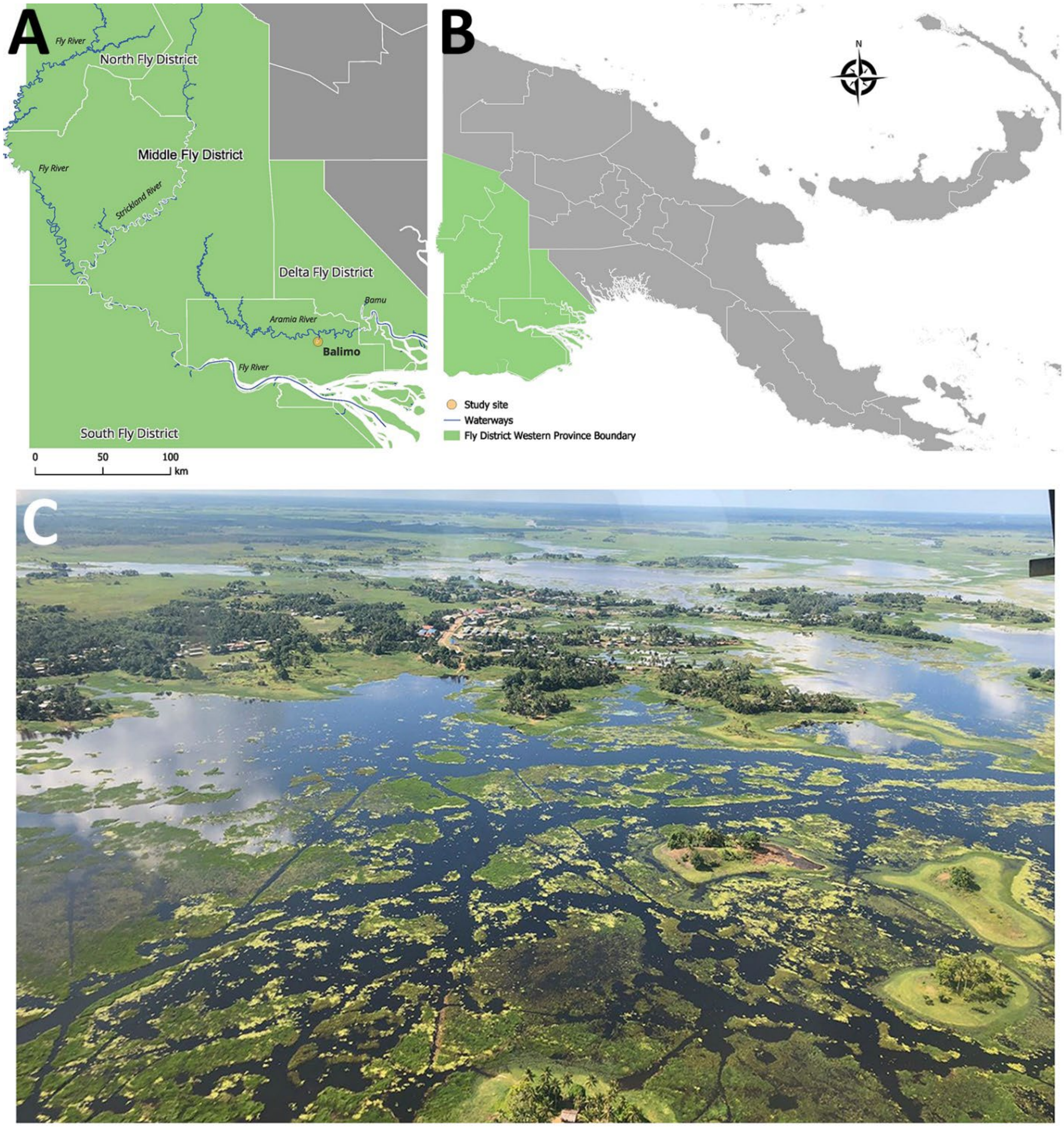

Appendix Figure 1. A, B) Western Province of Papua New Guinea showing the location of the study area, Balimo. C) Balimo township. Photograph by J.W., used with permission. Map created using the free and open-source QGIS version 3.34.12-Prizren; Open Source Geospatial Foundation, https://qgis.org. Administrative Boundary data from the National Statistics Office of Papua New Guinea © 2022, adapted under a Creative Commons Attribution-Noncommercial-ShareAlike 4.0 International License (CC BY-NC-SA 4.0). Waterway data © OpenStreetMap contributors, used under the Open Database License (ODbL; https://opendatacommons.org/licenses/odbl).

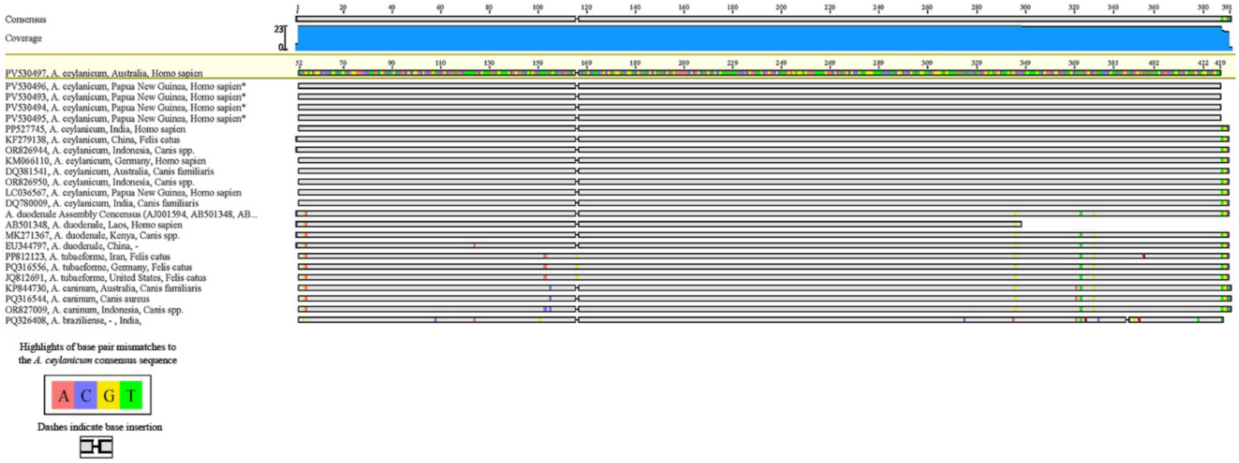

Appendix Figure 2. Sequence alignment of *Ancylostoma* species from GenBank and sequences found in this study. Alignment was mapped to the GenBank sequence PV530497, the positive *A. ceylanicum* DNA template control used in this study. Geneious version 2025.1 created by Biomatters (https://www.geneious.com).
